# Protective Effects of *Microsorum scolopendria* (Burm.f.) Copel. Leaf and Rhizome Extracts on Oxidative Stress and Inflammation Induced by *Staphylococcus aureus* and *Staphylococcus epidermidis*

**DOI:** 10.3390/antiox14101194

**Published:** 2025-09-30

**Authors:** Cristóbal Balada, Valentina Díaz, Mónica Castro, Macarena Echeverría-Bugueño, María José Marchant, Leda Guzmán

**Affiliations:** 1 Laboratorio de Biomedicina y Biocatálisis, Instituto de Química, Facultad de Ciencias, Pontificia Universidad Católica de Valparaíso, Avenida Universidad 330, Valparaíso 2340000, Chile; cristobal.balada@pucv.cl (C.B.); valentina.diaz@pucv.cl (V.D.); maria.marchant.l@pucv.cl (M.J.M.); 2 Laboratorio de Propagación, Escuela de Agronomía, Facultad de Ciencias Agronómicas y de los Alimentos, Pontificia Universidad Católica de Valparaíso, La Palma S/N, Quillota 2260000, Chile; monica.castro@pucv.cl; 3 Facultad de Ciencias de la Salud, Universidad de Las Américas, Sede Viña del Mar, 7 Nrte 1348, Viña del Mar 2340000, Chile; macarena.echeverria@edu.udla.cl

**Keywords:** COX-2, antioxidant activity, natural therapeutics, polyphenols

## Abstract

*Microsorum scolopendria* (Burm.f.) Copel. is a traditional medicinal fern with reported antioxidant and anti-inflammatory properties. In this study, we investigated the protective effects of leaf (HH) and rhizome (RH) extracts of *MS* on oxidative stress and inflammation in human dermal fibroblast (HDFa) cells infected with *Staphylococcus aureus* and *Staphylococcus epidermidis*. Cytotoxicity assays revealed that both extracts were safe up to 100 µg/mL, although RH exhibited a slight reduction in viability (≈20%) at 63 µg/mL. In infection assays, pretreatment with HH and RH extracts (63–100 µg/mL) for 3 h significantly reduced ROS levels by up to 45% compared with infected controls, while LDH release decreased by ~30%, indicating protection against membrane damage. Regarding anti-inflammatory activity, both extracts showed selective inhibition of COX-2 over COX-1, with RH inhibiting COX-2 by 62% and HH by 55% at 100 µg/mL, whereas COX-1 inhibition remained below 20%. These results highlight differential biological performance between leaf and rhizome extracts, with RH showing slightly higher anti-inflammatory activity but also a modest cytotoxic effect at intermediate concentrations. Overall, *MS* extracts demonstrated protective effects against oxidative and inflammatory damage induced by bacterial infection, supporting their potential as safe natural therapeutic agents for managing infection-associated skin stress and inflammation.

## 1. Introduction

*Microsorum scolopendria* (*MS*) is a fern native to tropical regions of Asia and the Pacific islands [1,2,3,4]. It belongs to the Polypodiaceae family and, as an epiphytic species, typically grows on trees, rocks, and other surfaces in humid forest environments [1,2,3,5]. MS has been traditionally used in some cultures for its medicinal properties [1,2,6], although scientific studies on its therapeutic potential remain limited. Extracts of *MS* have been reported to possess antioxidant activity, which may be attributable to the presence of polyphenols and other bioactive compounds [1,7]. Polyphenols are a class of naturally occurring compounds present in plants [1,8], characterized by the presence of multiple phenolic rings [2]. They are widely distributed across the plant kingdom, where they play significant roles in plant physiology and ecology [8,9].

In traditional medicine, *MS* has been widely used in different regions of the Pacific and Southeast Asia for a wide range of purposes. In Hawaii and Polynesia, it is used as an expectorant for the treatment of coughs, asthma, and bronchitis [10]. On five islands in French Polynesia, it is used as an infusion to treat restlessness, irritability, and jerking movements [11]. In Rapa Nui, the plant is considered part of the local medicinal heritage and is administered in oral or topical preparations to treat respiratory and skin conditions [1]. This diversity of ethnomedical uses supports the cultural and therapeutic importance of the species and motivates scientific validation of its effects.

Recent phytochemical studies have identified a wide range of secondary metabolites in the rhizomes and leaves of *MS*, including ecdysteroids, flavonoids, coumarins, stilbenes, psoralen, resveratrol, catechol, and 1,4-naphthoquinone, while cirsimaritin, protocatechuic acid 4-O-glucoside, and p-coumaroyl tartrate have been detected in rhizomes [12]. These compounds, well characterized for their antioxidant, antimicrobial, and anti-inflammatory properties, suggest that different parts of the plant concentrate chemical profiles with distinct therapeutic applications [13]. The identification of these metabolites is relevant because it connects ethnomedical use with molecular mechanisms potentially responsible for their effects.

Studies suggest that *MS* found in Rapa Nui may have potential as a source of natural antioxidants and antimicrobial agents [1]. However, further research is required to comprehensively understand the plant’s chemical composition and potential medicinal applications.

Polyphenols are well known for their antioxidant activity, which enables them to scavenge free radicals and protect macromolecules such as lipid, proteins, and DNA from oxidative damage [2,3,6]. These molecules also have anti-inflammatory, anti-cancer, and neuroprotective properties, among others [14,15]. They have also shown beneficial effects on epithelial damage in various parts of the body, like the skin. In fact, in the skin, the polyphenols have been shown to protect against epithelial damage caused by UV radiation, pollution, and other environmental stressors [1,16]. Epithelial damage can be caused by various factors, including microorganisms such as bacteria, viruses, fungi and parasites. The invasion of these microorganisms can induce inflammation and oxidative stress, which subsequently leads to the impairment of epithelial cells and barrier function [3,17].

Despite the available information, research on *MS* remains fragmentary and has significant gaps. Most studies are limited to preliminary chemical characterizations or partial evaluations of its bioactivity, without integrating functional assays in relevant cellular models. In particular, there is little evidence on the role of its extracts in modulating oxidative stress and the inflammatory response in human cells under bacterial infection, which limits understanding of its potential as a natural therapeutic agent.

## 2. Materials and Methods [18]

### 2.1. Plant Materials, Cells and Reagents

Plant specimens were collected from CONAF (Conservation Reserve on Rapa Nui). Upon arrival at the Laboratory of Biomedicine and Biocatalysis at Pontificia Universidad Católica de Valparaíso, rhizomes and leaves were weighed, cleaned with sterile water, chopped and stored at −80 °C until use. Adult Human Dermal Fibroblasts (HDFa) were obtained from Thermo Fisher Scientific (Waltham, MA, USA). The bacterial strains *S. aureus* (ATCC 25955) and *S. epidermidis* (ATCC 35984) were acquired from Microbiologics^®^ (MN, Warrington, PA, USA). The LDH Cytotoxicity Assay Kit was purchased from Thermo Fisher Scientific. Gallic acid, Trolox, AAPH, n-hexane, ethyl acetate, and DPPH were procured from Sigma-Aldrich (St. Louis, MO, USA). Culture medium 106, LSGS supplement, antibiotics (penicillin and streptomycin), Trypticase Soy Agar (TSA) and Trypticase Soy Broth (TSB) were obtained from Oxoid (Thermo Fisher Scientific, Waltham, MA, USA). All other reagents were purchased from Merck Co. (Kenilworth, NJ, USA).

### 2.2. Cell Growth Conditions

HDFa cells were maintained in medium 106 supplemented with LSGS, penicillin (50 U/mL) and streptomycin (50 µg/mL). Cells were incubated at 37 °C in a humidified 5% CO_2_ atmosphere. *S. aureus* and *S. epidermidis* were routinely cultured in TSA and TSB at 37 °C for 12–24 h. All strains were preserved at −80 °C in 15% glycerol.

### 2.3. Determination of MS Phytoextracts Metabolite Composition and Radical Scavenging Capacity

#### 2.3.1. Extract Preparation

Samples of 20 g of either leaves or rhizomes were washed with distilled water, dried, wrapped in absorbent paper and aluminum foil, and placed in an oven at 40 °C for 72 h. The dried material was then chopped and stored at −80 °C. Frozen samples were crushed, ground, and extracted with 500 mL of n-hexane under magnetic stirring at 35 °C for 72 h. Extracts were filtered using Whatman No. 1 filter paper, and hexane was evaporated using a rotary evaporator (Heidolph, Schwabach, Germany) at 40 °C. The dried extracts were dissolved in ethanol (10,000 µg mL^−1^) prior to storage at −20 °C, where they were kept for a maximum of four weeks before analysis. Ethanol was chosen as a solvent due to its ability to stabilize polyphenolic compounds, while also providing compatibility with in vitro assays. However, we recognize that extended storage in ethanol could promote partial degradation of some bioactive molecules, which represents a limitation of the present study.

#### 2.3.2. Determination of Total Phenolic Compounds

Total polyphenol content was quantified using the Folin–Ciocalteu reagent [18]. Extracts RH and HH were initially diluted 1:10 in water; then 200 µL of the diluted extract were added to the reaction mixture, resulting in a final concentration of 100 µg/mL. When the extracts were too concentrated, they were previously diluted in ethanol to ensure that the absorbance values fell within the calibration curve range. Subsequently, 125 µL of 1 N Folin–Ciocalteu reagent was added and mixed vigorously. Afterward, 625 µL of 20% Na_2_CO_3_ was added and the mixture was shaken for 2 h. Absorbance was measured at 760 nm using an HPUV 8453 spectrophotometer (Agilent, Santa Clara, CA, USA). Values were compared to a gallic acid standard curve (0–10 mg/L) and expressed as mg gallic acid equivalents (GAE)/g of dry extract. Experiments were performed in triplicate.

#### 2.3.3. Determination of Total Flavonoid Compounds

Total flavonoid content was determined following the method by Liu [19] with slight modifications. Briefly, 30 µL of 10% sodium nitrite, 60 µL of 20% aluminum chloride, 200 µL of 1 M NaOH, and 400 µL of distilled water were added to 100 µL of sample. After 5 min, absorbance was read at 415 nm. Concentrations were calculated using a kaempferol (KE) standard curve and expressed as KE/g dry extract. Experiments were performed in triplicate.

#### 2.3.4. Radical Scavenging Capacity Determined by the DPPH Assay

The antioxidant activity was measured by the DPPH assay [20]. A total of 1 mL of 0.1 mM DPPH in ethanol was mixed with 50 µL of RH or HH extract, gallic acid, TROLOX and Vitamin C (20 µg mL^−1^). The decrease in absorbance due to reduction of DPPH (purple to yellow) was recorded at 518 nm after 20 min using an Epoch ELISA reader (ELx800; BioTek, Winooski, VT, USA). Radical scavenging was calculated as:

% Inhibition = [(abs control − abs sample)/abs control] × 100(1)

The antioxidant capacity of the fractions using DPPH for free radical scavenging ability is expressed as IC_50_ of DPPH inhibition. The assay was performed in triplicate.

#### 2.3.5. ORAC-FL Assay

ORAC values were obtained following an established method [21] with modifications [18]. In sodium phosphate buffer (75 mM, pH 7.4), 20 µL of each extract or Trolox (20 µg mL^−1^) and 120 µL of fluorescein (70 nM final) were added to 96-well black plates and preincubated for 15 min at 37 °C. Then, 60 µL of AAPH (12 mM final) was added, and fluorescence was recorded every minute for 80 min (Synergy HT plate reader, BioTek). AUC was calculated as:

AUC = 1 + i = 80 ∑ i = 1 fi f0(2)

Net AUC was obtained by subtracting the blank, and results were expressed as Trolox equivalents. All experiments were performed in triplicate.

#### 2.3.6. RP-HPLC-MS/MS Analysis of *Microsorum scolopendria* Extracts

The analysis of the *MS* extracts was carried out using an Agilent HPLC 1100 system (Agilent, Santa Clara, CA, USA) coupled to a TRAP 3200 Q TRAP hybrid triple quadrupole/linear ion trap mass spectrometer. A binary solvent system was employed, consisting of solvent A (0.1% formic acid in water) and solvent B (100% methanol), with a flow rate set at 0.5 mL/min. Solvent B was increased from 5% to 50% over 30 min, followed by a further increase to 75% over 25 min. Mass detection was performed over a range of *m*/*z* 100–1000 in both positive and negative ionization modes. The resulting data were processed using Thermo Xcalibur SP1.48 software (version 2.2, Thermo Fisher Scientific, Waltham, MA, USA) and compound identification was assisted by molecular weight information obtained from the Phenol-Explorer database (version 3.6, http://phenol-explorer.eu/, accessed on 20 June 2021).

#### 2.3.7. Sun Protection Factor (SPF) Evaluation

Samples were diluted in ethanol (1 mg mL^−1^), filtered and scanned from 290 to 320 nm (5 nm intervals) using an HPUV 8453 spectrophotometer (Agilent) with a 1 cm quartz cuvette. SPF was calculated using the Mansur equation [22]:

SPF = CF × 320 ∑ 290 EE(λ) × I (λ) × Abs (λ)(3)

where EE is the erythemal effect spectrum, I correspond to the solar intensity spectrum, Abs is absorbance, and CF is a correction factor. Measurements were performed in triplicate.

### 2.4. Antimicrobial Assays

#### 2.4.1. Minimum Inhibitory Concentrations (MIC)

MIC_80_ values for both bacteria with and without *MS* extracts were determined. A 0.5 McFarland inoculum was added to Mueller Hinton Broth in 96-well plates with extract concentrations ranging from 0.060–512 µg/mL. Cultures were incubated at 37 °C, 150 rpm for 24 h. Growth was monitored by absorbance at 600 nm using an EPOCH spectrophotometer.

#### 2.4.2. Biofilm Formation Inhibition Assay

As described in [23], with modifications, a 0.5 McFarland standard was inoculated into Mueller Hinton Broth in 96-well plates. Bacteria were treated with MIC_80_ (final concentration in the well) and three serial dilutions. After incubation (37 °C, 150 rpm, 48 h), wells were washed twice with PBS, dried at 60 °C (1 h), stained with 0.4% crystal violet (15 min), washed, and dried again. Then, 200 µL of 0.03% acetic acid was added, and absorbance was measured at 570 nm. Results were compared to untreated controls. Measurements were performed in triplicate at three time points.

#### 2.4.3. Biofilm Disintegration Test

Following [23] with modifications, a 0.5 McFarland standard was cultured in Mueller Hinton Broth for 24 h at 37 °C, 150 rpm. Cultures were then treated with MIC_80_ (final concentration in the well) and three serial dilutions below MIC_80_ for 48 h. Biofilms were processed as above. Results were calculated relative to untreated controls. Measurements were performed in triplicate at three different times.

### 2.5. Cellular Assays

#### 2.5.1. Cytotoxicity Assay

The cytotoxic activity of *MS* extracts was evaluated using the immortalized human dermal fibroblast adult (HDFa) cell line, following the protocol described by Riss et al. [24]. Cells (3 × 10^3^ per well) were seeded into 96-well culture plates and incubated for 24 h with *MS* extracts in 106 media at 37 °C and 5% CO_2_.

Following the incubation period, cell viability was assessed using the MTS assay. This method is based on the reduction of the tetrazolium salt MTS by metabolically active cells, producing a soluble formazan product with a characteristic absorbance at 490 nm. A decrease in formazan production indicates reduced cell viability, suggesting a cytotoxic effect of the tested compounds.

#### 2.5.2. Evaluation of Cytotoxicity of *MS* Extracts in HDFa Cells During Infection with *Staphylococcus aureus* and *Staphylococcus epidermidis*

The cytotoxic effect of *MS* extracts was evaluated HDFa cells infected independently with *S. aureus* and *S. epidermidis* [1]. HDFa cells were seeded at a density of 2.7 × 10^3^ cells per well in 48-well plates and incubated for 24 h at 37 °C in 5% CO_2_ in 106 culture medium. Bacteria were added at a multiplicity of infection (MOI) of 5, meaning each fibroblast was exposed to five bacterial cells.

Cell viability was assessed by measuring the release of lactate dehydrogenase (LDH) using the LDH Cytotoxicity Detection Kit (TAKARA BIO, San José, CA, USA), which specifically quantifies LDH released from eukaryotic cells. Supernatants were collected at 3, 6, 12, and 24 h post-infection. The percentage of cytotoxicity was calculated using the following formula:

% Cytotoxicity = [(Treated cells − Untreated cells)/(Maximum damage control − Untreated cells)] × 100 (4)

#### 2.5.3. Measurement of Reactive Oxygen Species (ROS) Formation

To evaluate the generation of ROS, HDFa cells were seeded at a density of 5 × 10^3^ cells per well in a black-bottom treated 96-well plates and preincubated in culture medium for 24 h. Cells were then exposed to different concentrations of the tested extracts for 3 h, followed by infection with *S. aureus* or *S. epidermidis* (independently) for an additional 3 h. In parallel, co-treatment assays were performed in which both the extracts and bacteria were added simultaneously and incubated for 3 h.

After treatment, the culture medium was replaced with Krebs–Henseleit Buffer (KHB), and cells were incubated with 25 µM of the fluorescent probe H_2_DCFDA (2’,7’-dichlorodihydrofluorescein diacetate) for 30 min at 37 °C in the dark. ROS production was measured by monitoring the fluorescence of the oxidized DCF product (λexc/λem = 490/525 nm) using a fluorescence microplate reader (Thermo Scientific SkanIt^®^ Appliskan, Waltham, MA, USA). Measurements were taken kinetically for 30 min at 37 °C.

### 2.6. Inhibition of COX Enzymes

COX inhibition was assessed using BioVision^®^ fluorometric kits for COX-1 and COX-2 per manufacturer instructions. The inhibition of prostaglandin G2 formation from arachidonic acid was measured. Reactions included 3 µg mL^−1^ *MS* extract and SC560 (reference inhibitor) and were incubated at 25 °C for 10 min. Fluorescence (λexc/λem: 535/587 nm) was read using a SkanIt Appliskan plate reader (Thermo Fisher Scientific). Percentage inhibition was calculated as:

% Inhibition = slope Enzyme control − slope inhibitor compounds slope enzyme control × 100(5)

### 2.7. Statistical Analysis

All data are expressed as the mean ± standard deviation (SD). Differences between experimental groups were analyzed using Student’s *t*-test and one-way analysis of variance (ANOVA), followed by Tukey’s post hoc test, according to the experimental design. Differences were considered statistically significant at *p* < 0.001. Statistical analyses were performed using SPSS software (version 17; SPSS Inc., Chicago, IL, USA) and GraphPad Prism 10 (GraphPad Software Inc., La Jolla, CA, USA).

## 3. Results

### 3.1. Characterization of the Principal Chemical Components in Hexane MS Extracts

#### 3.1.1. Extraction Yield and Secondary Metabolites in Hexane Extracts

Table 1 presents the dry extract yield (mg g^−1^) obtained from *MS* HH and RH.

The total polyphenol and flavonoid contents were determined using the Folin–Ciocalteu and aluminum chloride methods, respectively. GAE and kaempferol (KE) were used as standards. Results are expressed as mg GAE g^−1^ and mg KE g^−1^ of dry sample. This information as shown in Table 2.

#### 3.1.2. RP-HPLC-MS/MS Profiling

**Figure 1:**
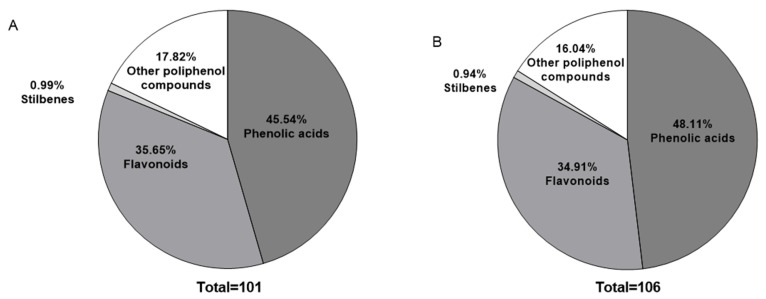
Relative distribution of polyphenol families in *MS* extracts from (**A**) RH, (**B**) HH (unit: %).

Polyphenolic compounds were identified using reversed-phase high-performance liquid chromatography coupled with tandem mass spectrometry (RP-HPLC-MS/MS), in both positive and negative ion modes. Figure 1 shows the distribution of polyphenol families in each extract, based on molecular weight matching using the Phenol-Explorer database. The principal polyphenols are shown in Table 3.

The percentages of polyphenol families present in the extracts were determined using molecular weights of over 1200 samples from the Phenol-Explorer database (Materials and Methods), as shown in Figure 1.

The phenolic acid family showed the highest relative abundance in both extracts. Flavonoids, stilbenes, coumarins and other polyphenol derivatives were also detected. Molecular weight ranges and glycosylation status of the compounds were evaluated (see Supplementary Materials Tables S1–S8).

### 3.2. Antioxidant Activity

Table 4 summarizes the antioxidant activity of the hexane extracts obtained from the rhizome (RH) and leaves (HH) of *MS*. Activities were evaluated by DPPH and the ORAC-FL method, as indicated in Materials and Methods.

The in vitro Sun Protection Factor (SPF) was calculated using the Mansur equation and the SPF values to RH and HH extracts are shown in Table 5.

### 3.3. Antimicrobial Assays

#### 3.3.1. Determination of the Minimum Inhibitory Concentrations (MIC)

The MIC of *MS* extracts against *S. aureus* and *S. epidermidis* were determined using Müller-Hinton broth. Kanamycin and chloramphenicol were used as positive antibiotic controls. The results are in Table 6.

The positive controls exhibited significantly lower MICs being classified as susceptible.

For *S. epidermidis*, HH showed a MIC_80_ of 32 µg mL^−1^ and an IC_50_ near 16 µg mL^−1^, while RH had a MIC_80_ of 256 µg mL^−1^ and IC_50_ around 32 µg mL^−1^. The MIC_80_ for Kanamycin was >512 µg mL^−1^ (resistant), and for Chloramphenicol it was 16 µg mL^−1^ denimed as intermediate susceptibility.

According to CLSI guidelines (2019), both *S. aureus* and *S. epidermidis* strains are resistant to the *MS* extracts HH and RH under the tested conditions.

#### 3.3.2. Biofilm Inhibition and Disruption Assay

Biofilm formation inhibition and biofilm disruption assays were conducted on *S.s aureus* and *S. epidermidis* using *MS* extracts HH and RH.

**Figure 2:**
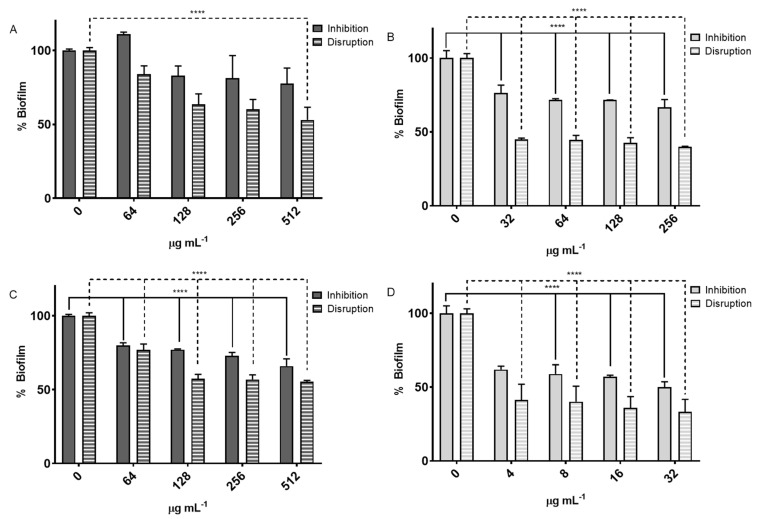
Biofilm formation inhibition and disruption assay for *S. aureus* and *S. epidermidis*. (**A**) RH treatment against *S. aureus.* (**B**) HH treatment against *S. aureus* (**C**) RH treatment against *S. epidermidis*. (**D**) HH treatment against *S. epidermidis*. **** indicates *p* < 0.0001 between no treatment control and treated bacteria.

For *S. aureus*, the RH extract demonstrated a notable impact on biofilm disruption, achieving a reduction of nearly 50%, whereas the HH extract resulted in approximately 40% disruption (Figure 2).

**Figure 3:**
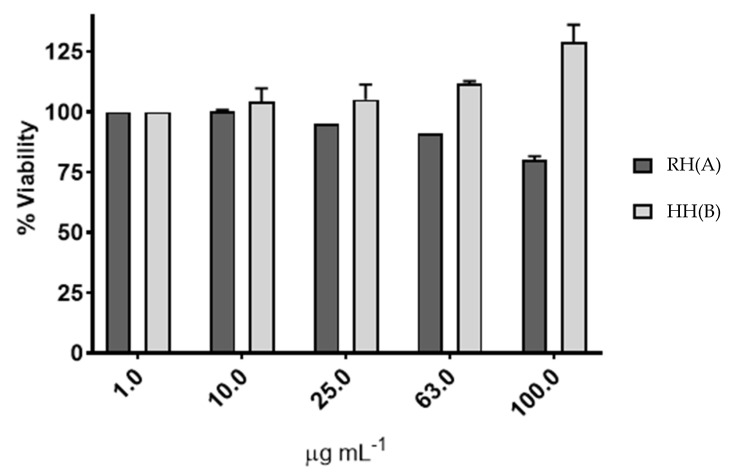
Viability of HDFa cells exposed to *Microsorum scolopendria* extracts over a concentration range from 1 µg mL^−1^ to 100 µg mL^−1^. (**A**) Viability of HDFa cells treated with RH for 24 h. (**B**) Viability of HDFa cells treated with HH for 24 h.

In *S. epidermidis*, HH extract exhibited significant effects on both the inhibition of biofilm formation, achieving approximately 32% at a concentration of 32 µg mL^−1^, and the disruption of existing biofilms. The RH extract demonstrated a greater impact on biofilm disruption compared to biofilm formation inhibition (Figure 3).

### 3.4. Cellular Assays

#### 3.4.1. Cytotoxicity Assay

To evaluate potential cytotoxicity, the effects of *MS* extracts RH and HH were assessed on the HDFa. The results indicated that only the RH extract exhibited slight cytotoxicity (Figure 3). Specifically, at a concentration of 63 µg mL^−1^, RH reduced cell viability by 19.91%. In contrast, the HH extract showed no cytotoxic effects at any of the tested concentrations.

Based on these results, a non-cytotoxic concentration range was determined for subsequent infection assays on HDFa cells. The selected concentrations were 25 and 63 µg mL^−1^ for RH, and 63 and 100 µg mL^−1^ for HH. These concentrations were chosen to ensure that the extracts would impact the bacterial pathogens without causing harm to human cells, supporting their suitability for topical application.

#### 3.4.2. Cytotoxicity of MS Extracts on HDFa Cells During Infection with *Staphylococcus aureus* and *Staphylococcus epidermidis*

**Figure 4:**
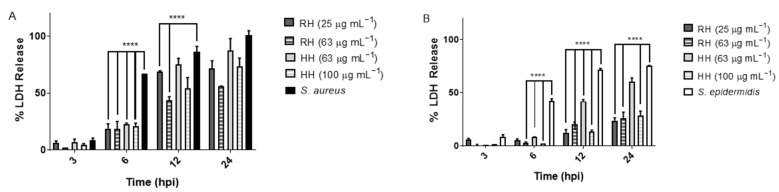
Effect of *M. scolopendria* on LDH Release in Infected HDFa Cells. The cells were infected with *S. aureus* (MOI 5) and treated with different concentrations of *MS* extracts and the release of LDH was measured. (**A**) Cells treated with RH. (**B**) Cells treated with HH. **** indicates *p* ≤ 0.0001 compared to the respective infected control without the extract.

To determine whether *MS* extracts RH and HH could mitigate host cell damage caused by a bacterial infection, HDFa cells were infected with *Staphylococcus aureus* or *Staphylococcus epidermidis* (MOI = 5) in the presence or absence of the extracts (Figure 4). After 24 h, the cytotoxicity was evaluated by measuring LDH release into the culture medium.

#### 3.4.3. Evaluation of Reactive Oxygen Species (ROS) Formation in HDFa Cell Line

##### Antioxidant Effect of *M. scolopendria* Extracts on ROS Levels

**Figure 5:**
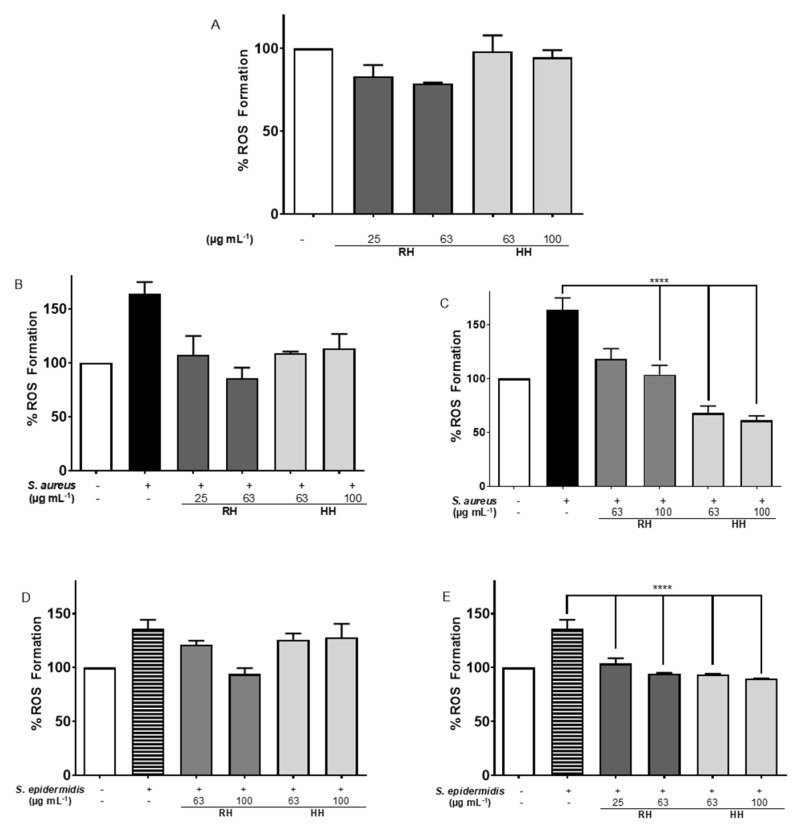
Percentage of reactive oxygen species. The bars correspond to the following: white for untreated cells, black for cells treated with *S. aureus*, black with stripes for cells treated with *S. epidermidis*, dark gray for cells treated with RH and the corresponding microorganism in the figure, and light gray for cells treated with HH and the corresponding microorganism in the figure. (**A**) HDFa cells exposed to *MS* extracts for 3 h. (**B**) Simultaneous exposure for 3 h to *S. aureus* with different concentrations of *MS* extracts. (**C**) Cells were incubated with different concentrations of *MS* extracts for 3 h, and then infected with *S. aureus* and incubated for 3 h. (**D**) HDFa cells exposed for 3 h to *S. epidermidis* with different concentrations of *MS* extracts. (**E**) HDFa cells pretreated for 3 h with different concentrations of *MS* extracts and subsequently incubated for 3 h with *S. epidermidis*. **** indicates *p* ≤ 0.0001 between the respective control of untreated cells and the treated cells.

ROS formation was assessed in HDFa cells treated with RH and HH extracts for 3 h (Figure 5A). After 3 h of treatment, no significant difference was observed compared to untreated cells; however, basal ROS levels were reduced in all treatments, with RH at 63 µg mL^−1^ showing the most notable decrease (below 80% of basal ROS). Similarly, 24-h treatment with RH and HH extracts maintained this reduction in basal ROS, confirming the antioxidant potential of these extracts over prolonged exposure.

Infection with *S. aureus* resulted in a significant increase in ROS formation (Figure 5B). The treatment with HH extract at 63 µg mL^−1^ reduced intracellular ROS by 56.5%. When cells were pre-treated for 3 h with HH extract before infection, ROS production decreased by 60.3% (Figure 5C), indicating that *HH* has a protective effect against oxidative stress induced by *S. aureus*.

When HDFa cells were incubated with *S. epidermidis* and *MS* extracts for 3 h (Figure 5D), only RH at 63 µg mL^−1^ significantly decreased ROS formation by 41.97%. Pre-treatment for 3 h with RH and HH extracts before infection led to a significant reduction in ROS production (RH: 32.0% and 41.3%; HH: 41.9% and 45.9%, Figure 5E), indicating protective antioxidant effects against *S. epidermidis*.

### 3.5. Evaluation of COX Enzyme Inhibition

The inhibitory effects of *MS* extracts RH and HH on COX-1 and COX-2 enzymes were evaluated tested the IC_50_ values and the selectivity indices measurement are summarized in Table 7. RH showed an IC_50_ of 1.97 ± 0.04 µg mL^−1^ for COX-2 and 7.19 ± 0.46 µg mL^−1^ for COX-1, resulting in a selectivity index of 3.65. HH presented an IC_50_ of 6.19 ± 0.61 µg/mL for COX-2 and 36.89 ± 4.96 µg mL^−1^ for COX-1, with a selectivity index of 5.96.

Both extracts displayed selective inhibition towards COX-2 over COX-1, which is therapeutically favorable. The selectivity indices were above 1 for both extracts, indicating preferential inhibition of the inducible COX-2 enzyme linked to inflammation, while sparing the constitutive COX-1 enzyme.

## 4. Discussion

### 4.1. Secondary Metabolite Content and Extraction Efficiency

The extraction yields show that the HH extract from *MS* leaves contains a higher amount of bioactive compounds compared to the RH extract from rhizomes, with nearly double the yield. This is consistent with previous reports indicating that hexane extracts from leaves often contain greater concentrations of surface-associated metabolites such as waxes, lipids, and terpenoids [25,26]. Moreover, leaves generally exhibit higher metabolic activity and can accumulate a more diverse and abundant array of secondary metabolites than subterranean structures such as rhizomes [27,28]. Quantification of total polyphenols and flavonoids revealed that HH extracts contained nearly twice the polyphenol content and almost four times the flavonoid content compared to RH extracts. These findings suggest a substantial enrichment of bioactive phenolic compounds in the leaf extracts. Notably, flavonoids represented more than 70% of the total phenolic content in the HH sample, highlighting their predominant contribution to its antioxidant profile. Similar results have been reported in other fern species, such as *Diplazium esculentum* and *Stenochlaena palustris* [29,30]. The high flavonoid concentration also supports previous qualitative reports of abundant flavonoids and tannins in *MS* [6]; however, this study provides the first quantitative confirmation for samples collected from Rapa Nui, thus complementing the limited existing data [1].

### 4.2. Identification and Profiling of Phenolic Compounds by RP-HPLC-MS/MS

Chromatographic profiling confirmed that phenolic acids are the predominant class of polyphenol in both extracts, accounting for over 45% of the total identified phenolics. These compounds, particularly hydroxybenzoic and hydroxycinnamic acid derivatives, are well known for their antioxidant and anti-inflammatory properties and have been widely reported in ferns [31,32,33]. In this study, the distribution between hydroxybenzoic and hydroxycinnamic acids was nearly equivalent (44% each), underscoring the chemical richness of *MS* as a source of these bioactives.

The flavonoid profile consisted primarily of glycosylated derivatives of common flavanols such as luteolin, kaempferol, and isorhamnetin. Kaempferol has previously been reported in *MS* species [34]. The average molecular weight of flavonoids (345 g mol^−1^), along with their core structures (~222 g mol^−1^), indicates a predominance of mono- and diglycosylated form, consistent with bioactive flavonoid profiles found in edible and medicinal ferns [35,36].

A notable observation was the high relative abundances of psoralen in both extracts, particularly in RH (47.25%) and HH (26.08%). Psoralen is a furanocoumarin reported to have phototoxic and therapeutic properties, including use in vitiligo treatment [37]. Its relative prominence in the hexane extracts may reflect adaptive responses of the plant to UV-rich environments or stress conditions. Other detected compounds, such as cirsimaritin, catechol, and resveratrol, also contribute to the potential bioactivity of the extracts. Although resveratrol was present at low relative abundance, its detection is notable due to its known antioxidant activity and broad therapeutic potential [35]. Additionally, pyrogallol in RH and 1,4-naphthoquinone in HH were identified, further illustrating the chemical diversity of *MS*.

It is important to emphasize that the identities of all detected compounds are tentative and based on LC-MS/MS data without confirmatory structural analysis or absolute quantification. The reported values reflect relative abundances, not absolute concentrations.

### 4.3. Antioxidant Capacity of Extracts

The antioxidant capacity of the hexane extracts from *MS* was found to be moderate compared to standard antioxidants. The RH extract exhibited greater DPPH radical scavenging activity, with a lower IC_50_ value (39.69 µg·mL^−1^) compared to the HH extract (68.35 µg·mL^−1^), which aligns with its higher content of polyphenols and flavonoids. However, both hexane extracts showed significantly lower activity than the reference antioxidants gallic acid and vitamin C. This limited antioxidant capacity is likely due to the predominance of lipophilic compounds in hexane extracts, which are generally less effective in hydrogen atom transfer (HAT) and single electron transfer (SET) mechanisms assessed by the DPPH assay, compared to more polar phenolics [38]. Therefore, while the HH extracts contain bioactive constituents, their radical scavenging potential may be underestimated by this assay, highlighting the need for complementary antioxidant evaluations using lipid-based or cell-based systems.

In the ORAC-FL assay, which measures scavenging capacity against peroxyl radicals, the HH extract exhibited a slightly higher antioxidant value (1.19) than the RH (0.91), suggesting that leaf-derived non-polar compounds may possess greater hydrogen atom transfer capacity. Both extracts displayed ORAC values comparable to the TROLOX standard.

The in vitro SPF values further reflect the modest antioxidant performance of these extracts. The HH extract achieved an SPF of 11.49, corresponding to a moderate-low classification, while the RH showed an SPF of 5.25, falling into the low category. These results indicate that although both extracts possess measurable antioxidant and photoprotective effects, their standalone application may be limited unless combined with other bioactive fractions or enriched extracts.

Overall, while the antioxidant potential of the HH and RH hexane extracts is moderate, their bioactivity supports their potential role as complementary ingredients in dermo-cosmetic or nutraceutical formulations.

### 4.4. Antimicrobial Activity

#### 4.4.1. MIC

The MIC results demonstrate that *MS* extracts possess limited antibacterial activity against *Staphylococcus aureus* and *Staphylococcus epidermidis*. Both extracts exhibited MIC_80_ values exceeding 512 µg mL^−1^ for *S. aureus*, indicating resistance, though the HH extract showed a 50% reduction in bacterial viability at 64 µg mL^−1^.

The antibiotic controls confirmed expected susceptibilities: Kanamycin and Chloramphenicol were highly effective against *S. aureus*, with MICs of 2 and 4 µg mL^−1^, respectively, indicating their suitability as positive controls. In contrast, *S. epidermidis* showed resistance to Kanamycin (>512 µg mL^−1^) but intermediate susceptibility to Chloramphenicol (16 µg mL^−1^), aligning with previous reports of variable antibiotic resistance profiles between staphylococcal species.

Against *S. epidermidis*, HH extract demonstrated greater antibacterial activity than RH, with a lower MIC_80_ of 32 µg mL^−1^ versus 256 µg mL^−1^. This indicates that *S. epidermidis* is more sensitive to the hexane leaf extract (HH) than to the rhizome extract (RH). The difference in antimicrobial potency could be related to distinct bioactive compounds present in the extracts, potentially including non-polyphenolic metabolites.

The relatively high MIC values for the *MS* extracts compared to the antibiotics suggest limited practical antibacterial utility in their crude form. However, the partial inhibition observed with HH extract on *S. epidermidis* may warrant further phytochemical fractionation to isolate and identify more potent antibacterial constituents.

Furthermore, these findings reinforce that total polyphenol content is not directly predictive of antibacterial activity, as the RH extract, likely richer in polyphenols, was less effective than HH. Future work should focus on detailed chemical profiling and synergistic testing with conventional antibiotics to enhance antibacterial effects.

#### 4.4.2. Biofilm Inhibition and Disruption Assay

A greater reduction in biofilm percentage was observed during the inhibition assay, which can be explained by the biofilm formation process consisting of four stages: (i) initial bacterial adhesion to the surface, (ii) microcolony formation, (iii) biofilm maturation, and (iv) bacterial dispersion to colonize new surfaces [39]. In the biofilm inhibition assay, bacteria are primarily in stages (i) and (ii), where intervention is more effective. In contrast, in the biofilm disruption assay, bacteria are already in stage (iii), where a mature biofilm structure forms a protective barrier that acts as a defense mechanism, making its removal more challenging. Both extracts exhibited a dose-dependent effect on biofilm formation inhibition, with greater reduction at higher concentrations, although the maximum inhibition was lower than that observed for disruption.

The HH and RH extracts of *MS* exhibited significant effects on biofilm formation and disruption in *S. aureus* and *S. epidermidis*, despite lacking significant bactericidal activity against *S. aureus*. This suggests that these extracts modulate bacterial virulence rather than directly killing the bacteria. Phenolic compounds, abundant in these extracts, have been reported to have limited impact on bacterial viability but play a crucial role in reducing virulence by interfering with quorum sensing mechanisms, decreasing the production of α-toxin family proteins, inactivating bacterial metabolism, reducing adhesion to surfaces, and inhibiting biofilm formation [40,41,42,43]. In this study, HH and RH extracts, which contain high phenolic content, were able to reduce both biofilm formation and stability, suggesting a diminished capacity for bacterial colonization and persistence.

The greater effect observed on biofilm disruption compared to inhibition may be explained by the structural complexity of mature biofilms, which act as physical and chemical barriers against external agents. The ability of these extracts to affect mature biofilms is promising for developing complementary therapies that enhance the efficacy of conventional antibiotics.

### 4.5. Cellular Assays

#### 4.5.1. Cytotoxicity Assay

The evaluation of cytotoxicity is a critical step in validating the potential therapeutic application of plant-derived extracts. In this study, *MS* extracts RH and HH were tested on HDFa fibroblasts to assess their safety for use in epithelial systems. The RH extract at 63 µg/mL reduced cell viability by approximately 20%, while no cytotoxicity was observed with the HH extract, even at the highest concentration tested (100 µg/mL), indicating that both extracts can be used safely within the tested concentration range. The moderate reduction in viability observed for RH does not preclude its potential use, especially since the effect was concentration-dependent and remained below the 30% cytotoxicity threshold generally considered acceptable for natural compounds in topical applications [44,45]. Importantly, both extracts allowed for the definition of safe, non-cytotoxic concentration ranges to be employed in subsequent bacterial infection assays, ensuring that any observed antibacterial or anti-inflammatory effects are not due to direct cytotoxicity on host cells. These findings are consistent with previous reports highlighting that some plant phenolic compounds may exert mild cytotoxic effects at higher concentrations, often linked to their oxidative or pro-oxidative properties in specific cellular environments. Nevertheless, the lack of significant cytotoxicity for HH, along with its previously observed bioactivities, underscores its potential as a safe candidate for further development in antimicrobial and anti-inflammatory therapies targeting skin infections.

#### 4.5.2. Cytotoxicity of *MS* Extracts on HDFa Cells During Infection with *S. aureus* and *S. epidermidis*

The protective effect of *MS* extracts on HDFa cells during infection with *S. aureus* and *S. epidermidis* was demonstrated through the reduction in LDH release. The significant cytotoxicity observed in *S. aureus*-infected cells, with nearly complete LDH release after 16 h, was markedly reduced when cells were co-treated with RH or HH extracts, especially at higher concentrations. This result suggests that these extracts can mitigate the deleterious effects of *S. aureus* infection on host cells.

The HH extract at 100 µg mL^−1^ showed the most notable protective effect, reducing LDH release to approximately 50%, followed by RH at 63 µg mL^−1^. This attenuation of cytotoxicity may be attributed to the biological activity of polyphenolic compounds present in the extracts, which are known to interfere with bacterial virulence mechanisms such as quorum sensing, toxin production and host cell adhesion [40,41,42,43].

In assays involving *S. epidermidis*, a lower level of cytotoxicity was observed overall, consistent with previous findings that this species exhibits reduced virulence compared to *S. aureus* [45]. Nevertheless, the application of RH and HH extracts further reduced LDH release, indicating a protective or anti-infective role even in the context of milder bacterial challenge. The decrease in cell damage may reflect a dual mechanism: direct inhibition of bacterial growth (as supported by Table 6 and suppression of biofilm formation (Figure 2), which limits the ability of *S. epidermidis* to persist and damage host cells.

These findings support the hypothesis that *MS* extracts, particularly RH and HH, not only reduce bacterial proliferation but may also alter the infection environment, reducing the likelihood of biofilm establishment and associated damage. The sustained cell viability observed in MTS assays (Figure 3) further reinforces the cytoprotective potential of these extracts.

While LDH release was not completely abolished, suggesting that some level of infection-induced damage remains, the observed reduction is biologically significant. It highlights the potential of these natural extracts as adjunctive agents capable of modulating host–pathogen interactions and reducing inflammation or cytotoxicity during bacterial infections.

As shown in Figure 4, exposure to *S. aureus* resulted in nearly 100% LDH release after 16 h, indicating extensive cell death. However, the presence of RH or HH extracts reduced LDH release in a dose-dependent manner. Treatment with 100 µg mL^−1^ of the HH extract led to a 50% LDH release, suggesting significant cytoprotection. Similarly, RH at 63 µg mL^−1^ also lowered LDH release, though to a slightly lesser extent.

When the same assay was performed using *S. epidermidis*, the observed cytotoxicity was notably lower than with *S. aureus*, in agreement with previous reports [45]. As shown in Figure 4 LDH release was reduced in infected cells treated with RH or HH extracts, particularly after 24-h incubations. The RH extract at both concentrations and HH at 100 µg mL^−1^ showed the most notable effects. This reduction in LDH release is consistent with a decrease in bacterial proliferation, a reduction in biofilm formation (Figure 2), and potentially a less favorable environment for *S. epidermidis* growth.

Although LDH release was not completely abolished, indicating some degree of cellular damage, the results suggest that RH and HH extracts may exert a protective effect on host cells by limiting bacterial virulence and enhancing cell viability. These findings were also supported by MTS assays (Figure 3), which showed improved viability of HDFa cells under the same conditions.

#### 4.5.3. Evaluation of Reactive Oxygen Species (ROS) Formation in HDFa Cell Line

The antioxidant effects of *MS* extracts RH and HH on human dermal fibroblasts were evident at 3 h, reflected in the sustained reduction in basal ROS levels. This suggests the extracts’ ability to mitigate oxidative stress over time, which is relevant for their potential application in skin protection.

During *S. aureus* infection, HH extract significantly attenuated ROS production both during co-incubation and following pre-treatment, indicating its role in modulating host cell oxidative responses and possibly inhibiting bacterial mechanisms that elevate ROS. The sustained antioxidant effect after 24 h further supports its therapeutic potential.

Similarly, RH extract showed notable antioxidative activity, especially against *S. epidermidis*, a less virulent pathogen. The reduction in ROS following pre-treatment suggests these extracts may be prime cellular antioxidant defenses, potentially through the upregulation of enzymes like glutathione peroxidase or superoxide dismutase, or by inhibiting pro-oxidative enzymes such as COX-2. These results suggest an increased expression of antioxidant proteins such as glutathione peroxidase and superoxide dismutase [46], or the inhibition of pro-oxidant proteins such as COX-2. It has been previously reported that a wide variety of flavonoids and stilbenes can inhibit pro-oxidative pathways and the activity of the COX-2 enzyme [47,48].

Overall, RH and HH extracts exhibit promising protective effects against oxidative damage induced by bacterial pathogens in dermal cells, with efficacy observed both in acute and extended exposures. These findings support their potential as natural antioxidant agents in managing skin infections and inflammation.

### 4.6. Evaluation of COX Enzyme Inhibition

The effect of the extracts on the inhibition of COX-1 and COX-2 enzymes was evaluated. COX-1 is constitutively expressed and is considered a housekeeping protein, responsible for maintaining normal physiological functions. In contrast, COX-2 is an inducible enzyme whose expression is activated in response to tissue damage and inflammatory conditions. Inhibition of COX-1 may lead to side effects such as impaired platelet aggregation and the formation of melanomas, whereas COX-2 inhibition has therapeutic effects on inflammation-associated pain [49]. Therefore, a selective inhibition of the COX-2 enzyme over COX-1 is desirable.

RH extract, with a selectivity index of 3.65 and an IC_50_ for COX-2 close to that of the positive control Celecoxib, demonstrated a dual role: moderate antioxidant capacity and potent enzyme inhibition. This suggests that polyphenols in RH may exert anti-inflammatory effects mainly through enzyme inhibition, consequently reducing intracellular ROS during bacterial infection.

HH extract exhibited a higher selectivity index (5.96) and good antioxidant capacity in ORAC assays, indicating that its anti-inflammatory effects may involve both direct scavenging of reactive oxygen species and COX-2 inhibition. This is supported by the considerable ROS decrease observed after 3-h pre-incubation with HH extracts in infection models.

These findings are consistent with previous antioxidant assays (DPPH and ORAC) where RH, despite showing a comparatively lower overall antioxidant capacity, demonstrated a strong COX-2 inhibitory effect, correlating with significant reductions in ROS production in cell infection models (Figure 5). HH extract showed moderate COX-2 inhibition and good antioxidant capacity by ORAC, along with notable ROS suppression when pre-incubated with cells.

Together, these results suggest that RH and HH extracts mitigate inflammation and oxidative stress through complementary mechanisms—enzyme inhibition and antioxidant activity—highlighting their promise for therapeutic development in skin inflammation and infection contexts.

Despite the promising results, this study has several limitations. First, the work was conducted exclusively in vitro using HDFa cells, which may not fully represent in vivo physiological conditions. Second, the chemical profiling of *MS* extracts was based on relative abundances obtained by RP-HPLC-MS/MS, without absolute quantification or full structural confirmation of compounds. Third, only two types of bacterial pathogens were evaluated, limiting generalization to other microorganisms. Finally, the long-term stability and bioavailability of the extracts were not assessed, which could impact their practical therapeutic application. Future studies should address these aspects to strengthen the translational potential of these findings.

## 5. Conclusions

The *MS* extracts RH and HH demonstrate significant potential as bioactive agents with antioxidant, anti-inflammatory, and cytoprotective properties. Cytotoxicity was evaluated on human dermal fibroblast cells (HDFa). RH showed a moderate reduction in cell viability (~20%) at 63 µg/mL, whereas HH maintained cell viability across the tested concentrations, suggesting a favorable profile for use in subsequent biological assays. These results allowed the definition of safe, non-cytotoxic concentration ranges for further studies, ensuring that the observed effects are not influenced by direct cytotoxicity for HH extract. Mechanistically, both RH and HH selectively inhibited the COX-2 enzyme, which is upregulated during inflammation, while sparing COX-1, reducing the likelihood of side effects commonly associated with non-selective COX inhibitors. The RH extract exhibited a potent COX-2 inhibitory effect with a selectivity index comparable to clinically used inhibitors, despite having a moderate overall antioxidant capacity. Conversely, HH combined moderate COX-2 inhibition with higher antioxidant activity, suggesting complementary anti-inflammatory pathways through enzyme inhibition and direct radical scavenging.

Collectively, these results position RH and HH extracts from *MS* as promising candidates for developing novel interventions targeting oxidative stress and inflammation associated with bacterial skin infections. Their dual action in protecting dermal cells and modulating inflammatory enzymes could offer effective and safer alternatives for managing skin-related inflammatory conditions.

## Figures and Tables

**Table 1:** Weight of extracts from *MS* samples.

Sample	Wight of Extract Obtained (mg g^−1^)	% Yield
RH	1.24	0.04
HH	2.46	0.59

**Table 2:** Total polyphenols and flavonoids in *MS* extracts.

Sample	Total Polyphenols (mg GAE g^−1^ Dry Sample)	Total Flavonoids (mg KE g^−1^ Dry Sample)
RH	6.59 ± 0.43	2.53 ± 0.06
HH	12.79 ± 0.67	9.52 ± 0.67

Values are expressed as mean ± standard deviation.

**Table 3:** Relative abundances of compounds in *MS* extracts.

			Relative Abundance (%)	
Compound	Family	Mode	RH	HH
Protocatechuic acid 4-O-glucoside	Phenolic acid	+	0.49	NA
p-Coumaroyl tartaric acid		−	NA	1.46
Cirsimaritin	Flavonoid	−	8.66	2.97
Kaempferide		−	NA	1.08
Resveratrol	Stilbene	+	0.43	1.51
Pyrogallol	Others	+	10.54	NA
Catechol		+	0.88	1.27
1,4-Naphthoquinone		+	NA	1.18
Psoralen		+	47.25	26.08

NA: Not among the most abundant in the extract.

**Table 4:** Radical scavenging capacity of *MS* extracts by DPPH and ORAC assays.

Sample	DPPH (IC_50_ µg·mL^−1^)	ORAC Value
RH	39.69 ± 2.34	0.91 ± 0.12
HH	68.35 ± 3.67	1.19 ± 0.02
Gallic acid	26.32 ± 0.97	1.03 ± 0.14
Vitamin C	29.42 ± 2.17	0.52 ± 0.04
TROLOX	-	1.00

Values are expressed as mean ± standard deviation.

**Table 5:** SPF found in *MS* extracts.

Sample	SPF Found	Corresponding SPF	SPF Level
RH	5.25 ± 0.01	6<	Low
HH	11.49 ± 0.08	10	Moderate low

Values are expressed as mean ± standard deviation.

**Table 6:** Minimum 80% inhibitory concentrations of *MS* extracts in *S. aureus* and *S. epidermidis*.

	*S. aureus*		*S. epidermidis*	
Sample	MIC_80_ (µg mL^−1^)	Category	MIC_80_ (µg mL^−1^)	Category
RH	>512	Resistant	256	Resistant
HH	>512	Resistant	32	Resistant
Kanamycin	2	Susceptible	>512	Resistant
Chloramphenicol	4	Susceptible	16	Intermediate susceptibility

**Table 7:** IC_50_ and selectivity indices of *MS* extracts on COX enzymes.

	IC_50_ (µg/mL)		
Sample	COX-1	COX-2	Selectivity Index
RH	7.19 ± 0.46	1.97 ± 0.04	3.65
HH	36.89 ± 4.96	6.19 ± 0.61	5.96
SC560	6.54 × 10^−3^ ± 9.02 × 10^−5^	-	-
Celecoxib	-	1.81 ± 0.02	-

Values are expressed as mean ± standard deviation.

## Data Availability

The original contributions presented in the study are included in the article and Supplementary Material, further inquiries can be directed to the corresponding author.

## Supplementary Materials

The following supporting information can be downloaded at: https://www.mdpi.com/article/10.3390/antiox14101194/s1, Table S1. Phenolic acids found in *MS* by RP-HPLC-MS/MS in negative mode. Table S2. Phenolic acids found in *MS* by RP-HPLC-MS/MS in positive mode. Table S3. Flavonoids found in *MS* by RP-HPLC-MS/MS in negative mode. Table S4. Flavonoids found in *MS* by RP-HPLC-MS/MS in positive mode. Table S5. Stilbenes found in *MS* by RP-HPLC-MS/MS in negative mode. Table S6. Stilbenes found in *MS* by RP-HPLC-MS/MS in positive mode. Table S7. Other polyphenol compounds found in *MS* by RP-HPLC-MS/MS in negative mode. Table S8. Other polyphenol compounds found in *MS* by RP-HPLC-MS/MS in positive mode.
