# Supplementary material for: Protective Effects of Microsorum scolopendria (Burm.f.) Copel. Leaf and Rhizome Extracts on Oxidative Stress and Inflammation Induced by Staphylococcus aureus and Staphylococcus epidermidis
Source: Antioxidants (Basel). 2025 Sep 30;14(10):1194. doi: 10.3390/antiox14101194 (PMC12561396; doi:10.3390/antiox14101194)
Supplement: Supplementary file 1 [file antioxidants-14-01194-s001.zip › antioxidants-3861854-supplementary.pdf]

Table S1. Phenolic acids found in *MS* by RP-HPLC-MS/MS in negative mode.

					RH				HH			
**Name**	**Molecular Formula**	**Subclass**	**Expected Mass (m/z)**	**Expected RT**	**RT**	**RT Variation**	**Observed Mass (m/z)**	**Mass Variation**	**RT**	**RT Variation**	**Observed Mass (m/z)**	**Mass Variation**
24-Methyl cholesterol ferulate	C38H56O4	Hydroxycinnamic acid	576.85	0.77	NA	NA	NA	NA	0.76	0.01	578.87	-2.02
4-Glucogallic acid	C13H16O10	Hydroxybenzoic acid	332.26	20.92	20.92	0	332.25	0.01	20.90	0.02	332.25	0.01
Galloyl glucose	C13H16O10	Hydroxybenzoic acid	332.07	20.92	20.92	0	332.25	-0.18	20.90	0.02	332.25	-0.18
Ellagic acid glucoside	C20H16O13	Hydroxybenzoic acid	464.34	22.52	22.52	0	464.32	0.02	22.49	0.03	464.32	0.02
Protocatechuic acid 4-O-glucoside	C13H16O9	Hydroxybenzoic acid	316.26	22.29	22.93	-0.64	316.26	0	22.94	-0.65	316.26	0
p-Coumaroyl tartaric acid	C13H12O8	Hydroxycinnamic acid	296.23	23.55	23.55	0	296.23	0	23.55	0	296.23	0
Caffeoyl tartaric acid	C13H12O9	Hydroxycinnamic acid	312.23	23.85	23.85	0	312.23	0	23.85	0	312.23	0
Valoneic acid dilactone	C21H10O13	Hydroxybenzoic acid	470.29	24.04	24.04	0	470.29	0	NA	NA	NA	NA
Gallic acid 3-O-gallate	C14H10O9	Hydroxybenzoic acid	322.22	24.05	24.02	0.03	322.21	0.01	NA	NA	NA	NA
4-Hydroxybenzoic acid 4-O-glucoside	C13H16O8	Hydroxybenzoic acid	300.26	25.42	25.42	0	300.26	0	25.42	0	300.26	0
Ellagic acid acetyl arabinoside	C21H16O13	Hydroxybenzoic acid	476.34	25.59	25.59	0	476.35	-0.01	25.56	0.03	476.35	-0.01
Ellagic acid acetyl xyloside	C21H16O13	Hydroxybenzoic acid	476.34	25.59	25.59	0	476.35	-0.01	25.56	0.03	476.35	-0.01

NA: Not among the most abundant in the extract.

Table S2. Phenolic acids found in *MS* by RP-HPLC-MS/MS in positive mode.

					RH				HH			
**Name**	**Molecular Formula**	**Subclass**	**Expected Mass (m/z)**	**Expected RT**	**RT**	**RT Variation**	**Observed Mass (m/z)**	**Mass Variation**	**RT**	**RT Variation**	**Observed Mass (m/z)**	**Mass Variation**
Dihydrocaffeic acid	C9H10O4	Hydroxyphenylpropanoic acid	182.17	18.69	18.53	0.16	182.16	0.01	18.65	0.04	182.16	0.01
Homovanillic acid	C9H10O4	Hydroxyphenylacetic acid	182.17	18.69	18.53	0.16	182.16	0.01	18.65	0.04	182.16	0.01
Caffeic acid	C9H8O4	Hydroxycinnamic acid	180.16	18.75	18.58	0.17	180.15	0.01	18.55	0.20	180.15	0.01
Gallic acid 4-O-glucoside	C13H16O10	Hydroxybenzoic acid	332.26	20.92	21.02	-0.10	332.25	0.01	21.03	-0.11	332.25	0.01
Ellagic acid glucoside	C20H16O13	Hydroxybenzoic acid	464.34	22.52	22.29	0.23	464.32	0.02	22.36	0.16	464.32	0.02
p-Coumaroyl malic acid	C13H12O7	Hydroxycinnamic acid	280.23	22.56	22.61	-0.05	280.23	0	22.61	-0.05	280.24	-0.01
p-Coumaric acid	C9H8O3	Hydroxycinnamic acid	164.16	22.67	22.87	-0.20	164.15	0.01	22.62	0.05	164.15	0.01
5-O-Galloylquinic acid	C14H16O10	Hydroxybenzoic acid	344.27	22.93	23.10	-0.17	344.28	-0.01	23.06	-0.13	344.28	-0.01
Protocatechuic acid 4-O-glucoside	C13H16O9	Hydroxybenzoic acid	316.26	22.93	23.08	-0.15	316.26	0	23.14	-0.21	316.26	0
p-Coumaroyl tartaric acid	C13H12O8	Hydroxycinnamic acid	296.23	23.55	23.57	-0.02	296.23	0	23.54	0.01	296.23	0
2-Hydroxybenzoic acid	C7H6O3	Hydroxybenzoic acid	138.12	23.56	23.56	0	138.13	-0.01	23.77	-0.21	138.13	-0.01
3-Hydroxybenzoic acid	C7H6O3	Hydroxybenzoic acid	138.12	23.56	23.56	0	138.13	-0.01	23.77	-0.21	138.13	-0.01
4-Hydroxybenzoic acid	C7H6O3	Hydroxybenzoic acid	138.12	23.56	23.56	0	138.13	-0.01	23.77	-0.21	138.13	-0.01
Vanillic acid	C8H8O4	Hydroxybenzoic acid	168.14	23.84	23.89	-0.05	168.15	-0.01	23.75	0.09	168.15	-0.01
Caffeoyl tartaric acid	C13H12O9	Hydroxycinnamic acid	312.23	23.85	23.71	0.14	312.22	0.01	23.67	0.18	312.22	0.01
Sinapic acid	C11H12O5	Hydroxycinnamic acid	224.21	23.91	23.93	-0.02	224.21	0	23.80	0.11	224.21	0
Valoneic acid dilactone	C21H10O13	Hydroxybenzoic acid	470.29	24.04	23.98	0.06	470.29	0	NA	NA	NA	NA
Gallic acid 3-O-gallate	C14H10O9	Hydroxybenzoic acid	322.22	24.05	23.82	0.23	322.22	0	24.11	-0.06	322.22	0
4-Hydroxybenzoic acid 4-O-glucoside	C13H16O8	Hydroxybenzoic acid	300.26	25.42	25.48	-0.06	300.26	0	25.67	-0.25	300.26	0
Ellagic acid acetyl arabinoside	C21H16O13	Hydroxybenzoic acid	476.34	25.59	25.39	0.20	476.35	-0.01	NA	NA	NA	NA

Ellagic acid acetyl xyloside	C21H16O13	Hydroxybenzoic acid	476.34	25.59	25.39	0.20	476.35	-0.01	NA	NA	NA	NA
Caffeoyl aspartic acid	C13H13NO7	Hydroxycinnamic acid	295.24	26.37	26.44	-0.07	295.25	-0.01	26.26	0.11	295.25	-0.01
Hydrocaffeic acid	C9H8O5	Hydroxycinnamic acid	196.16	26.42	26.43	-0.01	196.15	0.01	26.20	0.22	196.15	0.01
Gallic acid	C7H6O5	Hydroxybenzoic acid	170.12	26.46	26.49	-0.03	170.12	0	26.54	-0.08	170.12	0
Ellagic acid	C14H6O8	Hydroxybenzoic acid	302.19	26.55	26.42	0.13	302.20	-0.01	26.39	0.16	302.20	-0.01
Avenanthramide 2P	C16H13NO5	Hydroxycinnamic acid	299.28	26.61	NA	NA	NA	NA	26.69	-0.08	299.27	0.01
Phloretic acid	C9H10O3	Hydroxyphenylpropanoic acid	166.18	26.70	NA	NA	NA	NA	26.77	-0.07	166.17	0.01
Methoxyphenylacetic acid	C9H10O3	Hydroxyphenylacetic acid	166.17	26.70	NA	NA	NA	NA	26.77	-0.07	166.17	0
Dihydro-p-coumaric acid	C9H10O3	Hydroxyphenylpropanoic acid	166.17	26.70	NA	NA	NA	NA	26.77	-0.07	166.17	0
Caffeic acid 4-O-glucoside	C15H18O9	Hydroxycinnamic acid	342.29	26.71	26.77	-0.06	342.31	-0.02	26.66	0.05	342.30	-0.01
Caffeoyl glucose	C15H18O9	Hydroxycinnamic acid	342.29	26.71	26.77	-0.06	342.31	-0.02	26.66	0.05	342.30	-0.01
p-Coumaric acid 4-O-glucoside	C15H18O8	Hydroxycinnamic acid	326.29	27.32	27.16	0.16	326.30	-0.01	27.28	0.04	326.31	-0.02
2,3-Dihydroxybenzoic acid	C7H6O4	Hydroxybenzoic acid	154.12	35.26	35.09	0.17	154.12	0	35.26	0	154.12	0
2,4-Dihydroxybenzoic acid	C7H6O4	Hydroxybenzoic acid	154.12	35.26	35.09	0.17	154.12	0	35.26	0	154.12	0
2,6-Dihydroxybenzoic acid	C7H6O4	Hydroxybenzoic acid	154.12	35.26	35.09	0.17	154.12	0	35.26	0	154.12	0
2,5-Dihydroxybenzoic acid	C7H6O4	Hydroxybenzoic acid	154.12	35.26	35.09	0.17	154.12	0	35.26	0	154.12	0
Protocatechuic acid	C7H6O4	Hydroxybenzoic acid	154.12	35.26	35.09	0.17	154.12	0	35.26	0	154.12	0

NA: Not among the most abundant in the extract.

Table S3. Flavonoids found in *MS* by RP-HPLC-MS/MS in negative mode.

					RH				HH			
**Name**	**Molecular Formula**	**Subclass**	**Expected Mass (m/z)**	**Expected RT**	**RT**	**RT Variation**	**Observed Mass (m/z)**	**Mass Variation**	**RT**	**RT Variation**	**Observed Mass (m/z)**	**Mass Variation**
Kaempferol	C15H10O6	Flavonols	286.23	20.99	20.99	0	286.24	-0.01	20.98	0.01	286.24	-0.01
Luteolin	C15H10O6	Flavone	286.23	20.99	20.99	0	286.24	-0.01	20.98	0.01	286.24	-0.01
Scutellarein	C15H10O6	Flavone	286.24	20.99	20.99	0	286.24	0	20.98	0.01	286.24	0
Cyanidin	C15H11O6	Anthocyanins	287.24	21.00	21.00	0	287.24	0	21.00	0	287.24	0
6-Hydroxyluteolin	C15H10O7	Flavone	302.23	21.13	21.13	0	302.24	-0.01	21.16	-0.03	302.24	-0.01
Apigenin 7-O-glucuronide	C21H18O11	Flavone	446.40	22.24	22.24	0	446.36	0.04	22.26	-0.02	446.36	0.04
Cirsimaritin	C17H14O6	Flavone	314.29	22.64	22.64	0	314.30	-0.01	22.63	0.01	314.29	0
Isorhamnetin	C16H12O7	Flavonols	316.26	22.29	22.93	-0.64	316.26	0	22.94	-0.65	316.26	0
Nepetin	C16H12O7	Flavone	316.26	22.29	22.93	-0.64	316.26	0	22.94	-0.65	316.26	0
Ramnetina	C16H12O7	Flavonols	316.26	22.29	22.93	-0.64	316.26	0	22.94	-0.65	316.26	0
Daidzin	C21H20O9	Isoflavonoid	416.38	24.32	24.32	0	416.37	0.01	24.30	0.02	416.37	0.01
Hispidulina	C16H12O6	Flavone	300.26	25.42	25.42	0	300.26	0	25.42	0	300.26	0
Peonidin	C16H13O6	Anthocyanins	301.27	25.42	25.42	0	301.27	0	25.44	-0.02	301.27	0
Kaempferide	C16H11O6	Flavonols	299.25	25.43	25.43	0	299.26	-0.01	25.43	0	299.26	-0.01
Kaempferol 3-O-glucuronide	C21H18O12	Flavonols	462.36	25.54	25.54	0	462.36	0	25.57	-0.03	462.36	0
Luteolin 7-O-glucuronide	C21H18O12	Flavone	462.36	25.54	25.54	0	462.36	0	25.57	-0.03	462.36	0
Isoxanthohumol	C21H22O5	Flavonone	354.39	25.83	25.83	0	354.39	0	25.92	-0.09	354.39	0
Xanthohumol	C21H22O5	Chalcone	354.39	25.83	25.83	0	354.39	0	25.92	-0.09	354.39	0
6,8-Dihydroxykaempferol	C15H10O8	Flavonols	318.24	26.59	26.59	0	318.24	0	NA	NA	NA	NA
Miricetin	C15H10O8	Flavonols	318.24	26.59	26.59	0	318.24	0	NA	NA	NA	NA
Morin	C15H10O7	Flavonols	302.24	27.38	27.38	0	302.22	0.02	27.34	0.04	302.22	0.02
6-Prenylnaringenin	C20H20O5	Flavonone	340.36	28.64	28.50	0.14	340.38	-0.02	28.73	-0.09	340.38	-0.02
8-Prenylnaringenin	C20H20O5	Flavonone	340.36	28.64	28.50	0.14	340.38	-0.02	28.73	-0.09	340.38	-0.02
Daidzein	C15H10O4	Isoflavonoid	254.34	29.85	29.92	-0.07	254.22	0.12	29.92	-0.07	254.22	0.12

NA: Not among the most abundant in the extract.

Table S4. Flavonoids found in *MS* by RP-HPLC-MS/MS in positive mode.

					RH				HH			
**Name**	**Molecular Formula**	**Subclass**	**Expected Mass (m/z)**	**Expected RT**	**RT**	**RT Variation**	**Observed Mass (m/z)**	**Mass Variation**	**RT**	**RT Variation**	**Observed Mass (m/z)**	**Mass Variation**
Kaempferol	C15H10O6	Flavonols	286.23	20.99	21.04	-0.05	286.24	-0.01	20.97	0.02	286.24	-0.01
Luteolin	C15H10O6	Flavone	286.23	20.99	21.04	-0.05	286.24	-0.01	20.97	0.02	286.24	-0.01
Scutellarein	C15H10O6	Flavone	286.24	20.99	21.04	-0.05	286.24	0	20.97	0.02	286.24	0
Cyanidin	C15H11O6	Anthocyanins	287.24	21.00	20.80	0.20	287.24	0	21.23	-0.23	287.24	0
6-Hydroxyluteolin	C15H10O7	Flavone	302.23	21.13	21.14	-0.01	302.23	0	21.01	0.12	302.23	0
Formononetin	C16H12O4	Isoflavonoid	268.26	21.86	NA	NA	NA	NA	21.88	-0.02	268.26	0
Cirsimaritin	C17H14O6	Flavone	314.29	22.64	NA	NA	NA	NA	22.45	0.19	314.28	0.01
Isorhamnetin	C16H12O7	Flavonols	316.26	22.93	23.08	-0.15	316.26	0	23.14	-0.21	316.26	0
Nepetin	C16H12O7	Flavone	316.26	22.93	23.08	-0.15	316.26	0	23.14	-0.21	316.26	0
Ramnetina	C16H12O7	Flavonols	316.26	22.93	23.08	-0.15	316.26	0	23.14	-0.21	316.26	0
Daidzin	C21H20O9	Isoflavonoid	416.38	24.32	24.28	0.04	416.37	0.01	24.27	0.05	416.37	0.01
Floretina	C15H14O5	Dihydrochalcone	274.27	24.92	NA	NA	NA	NA	25.07	-0.15	274.26	0.01
Eriodictyol	C15H12O6	Flavonone	288.25	24.96	24.74	0.22	288.25	0	24.96	0	288.25	0
Sakuranetin	C16H14O5	Flavonone	286.27	25.26	25.48	-0.22	286.27	0	25.41	-0.15	286.28	-0.01
Hispidulina	C16H12O6	Flavone	300.26	25.42	25.48	-0.06	300.26	0	25.67	-0.25	300.26	0
Peonidina	C16H13O6	Anthocyanins	301.27	25.42	NA	NA	NA	NA	25.43	-0.01	301.27	0
Kaempferide	C16H11O6	Flavonols	299.25	25.43	NA	NA	NA	NA	25.67	-0.24	299.26	-0.01
Pelargonidin 3-O-arabinoside	C20H19O9	Anthocyanins	403.36	25.52	25.47	0.05	403.36	0	25.37	0.15	403.36	0
Kaempferol 3-O-glucuronide	C21H18O12	Flavonols	462.36	25.54	25.44	0.10	462.37	-0.01	25.71	-0.17	462.37	-0.01
Luteolin 7-O-glucuronide	C21H18O12	Flavone	462.36	25.54	25.44	0.10	462.37	-0.01	25.71	-0.17	462.37	-0.01
Isoxanthohumol	C21H22O5	Flavonone	354.39	25.83	25.92	-0.09	354.41	-0.02	NA	NA	NA	NA
Xanthohumol	C21H22O5	Chalcone	354.39	25.83	25.92	-0.09	354.41	-0.02	NA	NA	NA	NA
Miricetin	C15H10O8	Flavonols	318.24	26.59	26.49	0.10	318.23	0.01	26.46	0.13	318.23	0.01
Delfinidin 3-O-arabinoside	C20H19O11	Anthocyanins	435.36	26.93	26.86	0.07	435.35	0.01	27.10	-0.17	435.35	0.01
Biochanin A	C16H12O5	Isoflavonoid	284.26	27.17	27.03	0.14	284.27	-0.01	27.08	0.09	284.27	-0.01

NA: Not among the most abundant in the extract.

Table S5. Stilbenes found in *MS* by RP-HPLC-MS/MS in negative mode.

Name	Molecular Formula	Subclass	Expected Mass (m/z)	Expected RT	RH				HH			
					RT	RT Variation	Observed Mass (m/z)	Mass Variation	RT	RT Variation	Observed Mass (m/z)	Mass Variation
Resveratrol	C14H12O3	Stilbene	228.24	23.83	23.83	0	228.24	0	NA	NA	NA	NA

NA: Not among the most abundant in the extract.

Table S6. Stilbenes found in *MS* by RP-HPLC-MS/MS in positive mode.

Name	Molecular Formula	Subclass	Expected Mass (m/z)	Expected RT	RH				HH			
					RT	RT Variation	Observed Mass (m/z)	Mass Variation	RT	RT Variation	Observed Mass (m/z)	Mass Variation
Resveratrol	C14H12O3	Stilbene	228.24	23.83	23.99	-0.16	228.23	0.01	23.7	0.13	228.23	0.01

NA: Not among the most abundant in the extract.

Table S7. Other polyphenol compounds found in *MS* by RP-HPLC-MS/MS in negative mode.

Name	Molecular Formula	Subclass	Expected Mass (m/z)	Expected RT	RH				HH			
					RT	RT Variation	Observed Mass (m/z)	Mass Variation	RT	RT Variation	Observed Mass (m/z)	Mass Variation
5-Heneicosenylresorcinol	C27H46O2	Alkylphenol	402.7	0.86	0.86	0	401.99	0.71	0.9	-0.04	401.99	0.71
Ácido carnósico	C20H28O4	Phenolic Terpene	332.43	20.89	20.89	0	332.42	0.01	NA	NA	NA	NA
Esculina	C15H16O9	Hydroxycoumarin	340.28	26.37	NA	NA	NA	NA	26.3	0.07	340.29	-0.01

NA: Not among the most abundant in the extract.

Table S8. Other polyphenol compounds found in *MS* by RP-HPLC-MS/MS in positive mode.

					RH				HH			
**Name**	**Molecular Formula**	**Subclass**	**Expected Mass (m/z)**	**Expected RT**	**RT (RH)**	**RT Variation (RH)**	**Observed Mass (m/z) (RH)**	**Mass Variation (RH)**	**RT (HH)**	**RT Variation (HH)**	**Observed Mass (m/z) (HH)**	**Mass Variation (HH)**
Psoraleno	C11H6O3	Furanocoumarin	186.16	1	0.9	0.1	186.17	-0.01	0.87	0.13	186.17	-0.01
Pirogalol	C6H6O3	Other Polyphenols	126.11	1.05	1.11	-0.06	126.11	0	1.27	-0.22	126.11	0
3,4-Dihydroxyphenylglycol	C8H10O4	Other Polyphenols	170.16	12.87	12.84	0.03	170.17	-0.01	12.96	-0.09	170.17	-0.01
Syringaldehyde	C9H10O4	Hydroxybenzaldehyde	182.17	18.69	18.53	0.16	182.16	0.01	18.65	0.04	182.16	0.01
4-Hydroxycoumarin	C9H6O3	Hydroxycoumarin	162.44	21.13	21.3	-0.17	162.14	0.3	21.09	0.04	162.14	0.3
3,4-DHPEA-EDA	C17H20O6	Tyrosol	320.34	23.47	23.45	0.02	320.32	0.02	NA	NA	NA	NA
Protocatechuic Aldehyde	C7H6O3	Hydroxybenzaldehyde	138.12	23.56	23.56	0	138.13	-0.01	23.77	-0.21	138.13	-0.01
1,4-Naphthoquinone	C10H6O2	Naphthoquinone	158.15	23.83	23.83	0	158.15	0	23.79	0.04	158.15	0
3-Methylcatechol	C7H8O2	Alkylphenol	124.14	24.89	24.84	0.05	124.12	0.02	24.99	-0.1	124.12	0.02
4-Methylcatechol	C7H8O2	Alkylphenol	124.14	24.89	24.84	0.05	124.12	0.02	24.99	-0.1	124.12	0.02
Guaiacol	C7H8O2	Methoxyphenol	124.14	24.89	24.84	0.05	124.12	0.02	24.99	-0.1	124.12	0.02
Phlorin	C12H16O8	Other Polyphenols	288.25	24.96	24.74	0.22	288.25	0	24.96	0	288.25	0
Esculetin	C9H6O4	Hydroxycoumarin	178.14	25	NA	NA	NA	NA	25.14	-0.14	178.13	0.01
Catechol	C6H6O2	Other Polyphenols	110.11	26.13	26.09	0.04	110.1	0.01	26.33	-0.2	110.1	0.01
Esculina	C15H16O9	Hydroxycoumarin	340.28	26.37	26.46	-0.09	340.28	0	26.44	-0.07	340.28	0
Gallic Aldehyde	C7H6O4	Hydroxybenzaldehyde	154.12	35.26	35.09	0.17	154.12	0	35.26	0	154.12	0
Isopimpinellin	C13H10O5	Furanocoumarin	246.21	47.38	47.36	0.02	246.21	0	47.37	0.01	246.21	0

NA: Not among the most abundant in the extract.
